# Overexpression of VviPGIP1 and NtCAD14 in Tobacco Screened Using Glycan Microarrays Reveals Cell Wall Reorganisation in the Absence of Fungal Infection

**DOI:** 10.3390/vaccines8030388

**Published:** 2020-07-15

**Authors:** Florent Weiller, Lorenz Gerber, Johan Trygg, Jonatan U. Fangel, William G.T. Willats, Azeddine Driouich, Melané A. Vivier, John P. Moore

**Affiliations:** 1 South African Grape and Wine Research Institute, Department of Viticulture and Oenology, Stellenbosch University, Stellenbosch 7602, South Africa; florent@sun.ac.za (F.W.); mav@sun.ac.za (M.A.V.); 2 Department of Plant Sciences, Swedish Agricultural University, 75007 Uppsala, Sweden; lorenzottogerber@gmail.com; 3 Computational Life Science Cluster, Department of Chemistry, University of Umeå, 901 87 Umea, Sweden; johan.trygg@umu.se; 4 Department of Plant and Environmental Sciences, University of Copenhagen, 1165 Copenhagen, Denmark; jonatanfangel@gmail.com; 5 School of Agriculture, Food and Rural Development, Newcastle University, Newcastle-upon-Tyne NE1 7RU, UK; william.willats@newcastle.ac.uk; 6 Laboratoire de Glycobiologie et Matrice Extracellulaire Végétale (GlycoMEV), University of Rouen, 76821 Mont Saint Aignan, France; Azeddine.Driouich@univ-rouen.fr

**Keywords:** cell wall, lignin, pectin, extensin, PGIP, CAD, tobacco

## Abstract

The expression of *Vitis vinifera* polygalacturonase inhibiting protein 1 (VviPGIP1) in *Nicotiana tabacum* has been linked to modifications at the cell wall level. Previous investigations have shown an upregulation of the lignin biosynthesis pathway and reorganisation of arabinoxyloglucan composition. This suggests cell wall tightening occurs, which may be linked to defence priming responses. The present study used a screening approach to test four VviPGIP1 and four NtCAD14 overexpressing transgenic lines for cell wall alterations. Overexpressing the tobacco-derived cinnamyl alcohol dehydrogenase (*NtCAD14*) gene is known to increase lignin biosynthesis and deposition. These lines, particularly PGIP1 expressing plants, have been shown to lead to a decrease in susceptibility towards grey rot fungus *Botrytis cinerea*. In this study the aim was to investigate the cell wall modulations that occurred prior to infection, which should highlight potential priming phenomena and phenotypes. Leaf lignin composition and relative concentration of constituent monolignols were evaluated using pyrolysis gas chromatography. Significant concentrations of lignin were deposited in the stems but not the leaves of NtCAD14 overexpressing plants. Furthermore, no significant changes in monolignol composition were found between transgenic and wild type plants. The polysaccharide modifications were quantified using gas chromatography (GC–MS) of constituent monosaccharides. The major leaf polysaccharide and cell wall protein components were evaluated using comprehensive microarray polymer profiling (CoMPP). The most significant changes appeared at the polysaccharide and protein level. The pectin fraction of the transgenic lines had subtle variations in patterning for methylesterification epitopes for both VviPGIP1 and NtCAD14 transgenic lines versus wild type. Pectin esterification levels have been linked to pathogen defence in the past. The most marked changes occurred in glycoprotein abundance for both the VviPGIP1 and NtCAD14 lines. Epitopes for arabinogalactan proteins (AGPs) and extensins were notably altered in transgenic NtCAD14 tobacco.

## 1. Introduction

Pathogens such as necrotrophic or biotrophic plant pathogenic fungi have to break through/disrupt the host cell wall before colonising and feeding can begin. The first line of defence against the invading organism is the plant cell wall, which is a ‘dynamic’ glycan-matrix, composed of pectins-phenolics-proteins and hemicellulose layers depending on the plant species [1]. The plant cell wall matrix of fruits is a co-evolved pectin-protein-phenolic matrix, which aids in both the protection and dispersal of encapsulated seeds. Plant cell wall composition can be very different between fruits, such as grapes containing cell walls rich in pectin, grasses (i.e., monocotyledon) with secondary cell walls composed of mainly xylan-abundant polymers, or root tissues that are rich in xylogalacturonans [1,2]. The plant is able through pathogen detection systems to then modify its wall composition in response to a perceived stressor [3]. Callose deposition, lignification, and pectin esterification alterations are common responses at local points of contact between the pathogen and plant [1,3]. Invariably, this must mean that the plant pathogen (e.g., fungus) must also develop a molecular toolkit of weapons, such as enzymes, which are able to overcome and degrade the cell wall to access the host organism [1,4]. In addition to polysaccharides, cell wall proteins such as extensins have been shown to be plant–pathogen response macromolecules with an important role for arabinosylation in cross-linking these proteins at the site of deposition presumably impeding infection [5,6]. Plant pathogens can also use mechanical force via an appressorium [7], or use a wide range of plant cell wall degrading enzymes (CWDEs) to a good effect [8,9] to overcome the plant cell wall barrier.

One of the first enzymes released, and essential to fungal pathogenicity, are endo-polygalacturonases (ePGs) [10,11] in common terminology. That is not to say for example that CWDEs such as ePGs are more important than pectate lyases or pectin methyl esterases (PMEs) [12,13], but it is clear that they have been studied for the longest time in plant defence. ePGs hydrolyse the O-glycosyl bonds of α-1,4-linked galacturonic acid residues that form polygalacturonan chains. To counter the action of these fungal enzymes as well as regulate the plants own cell wall remodelling enzymatic process [14], plants have proteins with inhibiting properties such as the polygalacturonase inhibiting proteins (PGIPs), a conserved leucine rich repeat protein with ePG inhibiting activity [15,16,17]. PGIPs seem to have evolved to protect plants against fungal pathogens. However, the conserved leucine rich repeat pattern of the protein also suggests that the protein has indirect signalling roles in plants and may no longer function in a direct inhibiting action [18]. In some cases, selected PGIPs might have evolved to such a degree to have functions completely removed from directly inhibiting ePGs but rather in modulating molecular signalling pathways by activating seemingly unrelated plant pathways.

Moving back to the well-established idea of a direct role for PGIPs in inhibiting ePGs activity [19] a clear mechanism is still lacking. Docking studies by Federici et al. [20] have shown that PGIP2 from *Phaseolus vulgaris* can inhibit *Fusarium moniliforme* PG1 by a ‘dual’ action. Sicilia et al. [21] also showed that inhibition of *Botrytis cinerea* PG1 (*Bc*PG1) was achievable using PGIP2 isolated from *Phaseolus vulgaris*. Liu et al. [22,23] also showed with docking studies that PGIP1 can interact with various ePGs with specific binding sites depending of the ePG. However, no in vitro interaction evidence could be detected between *Bc*PG2 and *Vitis vinifera* PGIP1 (*Vvi*PGIP1) despite a reduction of symptoms observed during *Nicotiana benthamiana* co-infiltration experiments [19,24]. It was further suggested that PGIPs can interact directly with pectins to protect them from the action of the fungal enzymes via a competitive interaction process [25]. PGIPs have also been thought to stimulate plant defence responses by favouring the accumulation of oligogalacturonides [26]. Oligogalacturonides (OGs) are α-1,4-galacturonic acid oligomers released after the action of ePGs on homogalacturonans (HG). These OGs function as damage-associated molecular patterns (DAMPs) and are therefore potentially capable of activating various plant defence responses [27,28,29,30,31].

VviPGIP1 may also be activating defence pathways indirectly of the enzyme-inhibitor protein direct interaction paradigm [17]. Overexpressing VviPGIP1 in a useful patho-system, *Nicotiana tabacum* (tobacco), resulted in changes in cell wall gene expression and lignin specific staining in leaves and stems of wild type compared to transgenic plants [18]. VviPGIP1 was also studied at the promoter level showing its regulation is inducible via defence response triggers and signaling pathways [32]. Constitutive expression of *Vvipgip1* in tobacco leads to alteration in phytohormone levels but also in some cell wall components [18]. One of the genes found to be altered in the microarray datasets was a cinnamyl alcohol dehydrogenase (CAD). CAD is a key enzyme in lignin biosynthesis, and indeed, when upregulated resulted in stem lignification of transgenic tobacco. In order to investigate a possible role for CAD upregulation in cell wall strengthening a number of transgenic lines of tobacco plants overexpressing NtCAD14 was generated [33]. However based on pathogen ‘challenge’ datasets, CAD activity alone appears insufficient to impart resistance to transgenic tobacco plants [33].

VviPGIP1 expression clearly influences polysaccharide remodelling. This was supported from microarray and additional cell wall datasets. Xyloglucan endotransglycosylase/hydrolase (XET/XTH) genes showed altered expression compared to wild types. The use of CoMPP (comprehensive microarray polymer profiling) with fractionation and enzymatic data focused on the changes found in the arabinoxyloglucan components of transgenic versus wild-type plants [18,34]. A model was proposed in Nguema-Ona et al. [34] where the plant became ‘primed’ due to VviPGIP1 expression, which produced a lignified and tightened xyloglucan network better able to resist infection by a fungal pathogen such as *Botrytis cinerea* [34]. More recently the role of xyloglucan as a new DAMP in plant–pathogen interactions in *Arabidopsis* and *V. vinifera* has been uncovered [35] supporting the Nguema-Ona et al. [34] study. Given a set of VviPGIP1 overexpressing lines are available and the CAD tobacco lines produced in the Mbewana [33] study, it was thought a screening study would be most useful and informative. In the CAD lines for example does the expression of the gene also lead to cell wall alterations in the absence of a pathogen? This for example remains unclear. Therefore, both the VviPGIP1 and CAD tobacco lines were investigated using pyrolysis gas chromatography mass spectrometry (Py-GC–MS) to more accurately dissect the lignin and phenolic differences/similarities. Using a survey screening approach, the variation in cell wall polysaccharide composition and organisation was investigated by performing CoMPP and monosaccharide analysis (GC–MS) of all available transgenic tobacco lines.

## 2. Materials and Methods

### 2.1. Plant Material

The transgenic tobacco lines used in this study were generated using *Nicotiana tabacum* cv Havana petit SR1 that either constitutively express the *VviPGIP1* gene as described in Joubert et al. [24], or the *NtCAD14* gene as described in Mbewana [33]. The *VviPGIP1* transformants and the CAD32, CAD38, and CAD42 lines did not show any phenotypical difference with the SR1 control. The CAD4 line had a delayed growth in the developmental time of the experiment though fully developed plants reach the equivalent size as SR1. See Table 1 for additional information.

### 2.2. Plant Growth Conditions

Seeds (wild type tobacco *Nicotiana tabacum* cv Havana petit SR1) were surface sterilised [37] and sown on MS medium [38]. Transgenic seeds [24,33] were sown under Kanamycin resistance. Seedlings were hardened off in peat moss plugs (Jiffy Products International, AS, Norway) before they were transferred into pots containing an equal mixture of soil, peat moss, and vermiculite. The plants were grown in a clima-room (artificial light intensity of 120 μmol.m^−2^.s^−1^) with a sixteen hours light and eight hours dark photoperiod at 23 °C. Organic fertilizer (Nitrosol, Fleuron (Pty) Ltd., South Africa) was added during watering once every two weeks. Plant material was harvested when six leaves had fully expanded (see Supplementary Materials Figure S1). Leaves were labelled leaf position 3 (L3) to leaf position 6 (L6) as outlined in previous studies such as Nguema-Ona et al. [34] and where leaf 1 is nearest to the shoot apical meristem. A biological sample consisted of three leaves at a specific leaf position (e.g., leaf 3) was harvested from four different plants at the same time and immersed into liquid nitrogen. Hence, the sampling was a form of biological composite representative sampling to ensure sufficient material was available for downstream analysis.

### 2.3. Cell Wall Isolation Protocol

The selected leaves were processed for alcohol insoluble residue (AIR) preparations as described in Nguema-Ona et al. [34,39]. Leaf material was plunge frozen with liquid nitrogen and then ground to a fine powder using a Retsch Mixer Mill (30 rounds per minute, 60 s, Retsch, Haan, Germany). After boiling the tissue powder for 20 min in 80% aqueous ethanol (reagent grade) to deactivate enzymes, the insoluble material was washed with methanol for two hours on a rotating wheel. After centrifugation at 3000 rpm for 3 min, the pellets were sequentially washed twice with a (1:1) methanol/chloroform solution for 2 h to remove plant oils/lipids and then rinsed with acetone for another 2 h before being air-dried. The extracted material was resuspended in distilled water and freeze-dried to obtain cell wall AIR. Samples were destarched using a combination of thermostable alpha-amylase, amyloglucosidase and pullulanase (from Megazyme). The AIR material was used for all further analytical experiments. All solvents used were from (Sigma-Aldrich, MO, USA) at reagent grade.

### 2.4. Pyrolysis Gas Chromatography Mass Spectrometry (Py-GC–MS) for Lignin Analysis

The AIR samples were analysed according to Gerber et al. [40]. Briefly, the analytical setup consisted of an oven pyrolyser equipped with an auto sampler (PY-2020iD and AS-1020E, Frontier-Labs, Japan) mounted on a GC–MS system (Agilent, 7890A/5975C, Agilent Technologies AB, Sweden). The pyrolysis oven was set to 450 °C, the interface to 340 °C and the injector to 320 °C. The injector was operated with a split ratio of 16:1, with helium as the carrier gas. The pyrolysate was separated on a DB-5MS capillary column (30 m × 0.25 mm i.d., 0.25 μm film thickness; J&W, Agilent Technologies AB, Sweden). The GC temperature program was as follows: the analysis was started at 40 °C and was then increased by 32 °C min^−1^ to 100 °C, followed by 6 °C min^−1^ to 120 °C, by 15 °C min^−1^ to 250 °C and finally by 32 °C min^−1^ to 320 °C where the temperature was kept for a further 3 min. The MS interface was kept at 280 °C. The quadrupole mass spectrometer (operated at unit mass resolution) recorded spectra in the range from 35 to 250 m/z. Quantification of lignin in particular was performed by calculating integrated peak areas from selected m/z channels.

### 2.5. Gas Chromatography–Mass Spectrometry (GC–MS) for Monosaccharides

Monosaccharide composition of the tobacco AIR isolated was determined using GC–MS according to the method of York et al. [41]. 5 mg of AIR was hydrolysed with 2 M TFA at 110 °C for 2 h. After centrifugation, the supernatant containing the monosaccharides was converted to methoxy sugars using an application of 1 M methanolic HCl at 80 °C for 16 h. Silylation was performed on the methoxy sugars by addition of a solution HMDS + TMCS + Pyridine (3:1:9; Sylon HTP kit; Sigma-Aldrich, MO, USA) and incubation at 80 °C for 20 min in order to produce trimethyl-silyl-glycosides. TMS glycosides were then dissolved in cyclohexane before being injected onto a Agilent 6890 N (Agilent, Palo Alto, CA, USA) gas chromatograph coupled to a Agilent 5975 MS mass spectrometer detector. Separation was performed on a polar (95% dimethylpolysiloxane) ZB-Semi-Volatiles Guardian (30 m, 0.25 mm ID, and 0.25 µm film thickness) 7HG-G027-11 GC column. The carrier gas was helium with a flow rate of 1 mL/min and the injector temperature was maintained at 280 °C in the splitless mode. The oven temperature program was maintained at 80 °C for 1 min and finally ramped at 7 °C/min to 300 °C and then held for 2 min. Mass spectral data was recorded in full scan mode (40–650 m/z) with both the ion source and quadrupole temperatures maintained at 240 °C and 150 °C respectively. The nine main monosaccharides found in plant cell walls were measured and quantified using standard sugar mixtures and myo-inositol as internal standard measured using Xcalibur software (Thermo Fisher Scientific Inc., MA, USA).

### 2.6. Comprehensive Microarray Polymer Profiling (CoMPP) for Polysaccharides-Proteins

Comprehensive microarray polymer profiling (CoMPP) was performed as described by Kračun et al. [42]. 10 mg of AIR was first subject to a chelating agent CDTA (diamino-cyclohexane-tetraacetic acid, 50 mM, pH 7.5) and then to an alkali extraction with NaOH (4 M + 0.1% NaBH_4_). The AIR was extracted in 300 µL for each extract by agitating the tubes (each tube contains a glass bead) first at a frequency of 30 Hz for 2 min followed by 6 Hz for 2 h. This was performed sequentially for each extract so a total of 600 µL was collected for the CDTA (soluble pectin) and 600 µL was collected for the NaOH (soluble pectin-hemicellulose) per 10 mg of AIR. CDTA and NaOH extracts were pipetted into 384-microwell plates. Each sample was prepared as 4 dilutions (first dilution: 1:1, followed by serial five-fold dilutions of the preceding sample). From the microwell plates all samples and dilutions were then printed at 55% humidity onto nitrocellulose membranes (Whatman, pore size of 0.45 mm Whatman) using a microarray robot (Sprint, Arrayjet, Roslin, UK). Printed arrays were blocked using phosphate buffered saline PBS (140 mM NaCl, 2.7 mM KCl, 10 mM Na_2_HPO4, 1.7 mM KH_2_PO4, and pH 7.5) with 5% (*w*/*v*) low fat milk powder for 1 h followed by an incubation of 2 h with the various plant cell wall specific probes selected. This was followed by secondary binding in PBS and for 2 h with anti-rat, anti-mouse, or anti-His tag antibodies conjugated to alkaline phosphatase (Sigma) diluted 1:5000 (anti-rat and anti-mouse) or 1:1500 (anti-His tag). Details about the antibodies used are available in Table 2. Arrays were washed in PBS and then developed in a solution containing 5-bromo-4-chloro-3-indolylphosphate and nitro blue tetrazolium in alkaline phosphatase buffer (100 mM NaCl, 5 mM MgCl_2_, 100 mM diethanolamine, and pH 9.5). Arrays were dried and antibodies/CBMs (Carbohydrate binding module) signal intensity was scanned at 2400 dots per inch (dpi) using a CanoScan 8800F instrument (Søborg, Denmark) and converted to TIFF image files. The pixel intensity reads representing probe signals were quantified using Array-Pro Analyzer 6.3 (Media Cybernetics, Rockville, MD, USA) software. The average of all 4 dilutions for each sample was calculated. Results data were presented in the heatmaps format with the maximal mean spot signal intensity set to 100 and other signal values normalised accordingly. A cut-off of 5 was applied as a baseline to remove the noise signal.

### 2.7. Univariate Statistical and Multivariate Data Analysis

Twelve plants were grown for each genotype. Leaves of the same developmental stage from three different plants were pooled together, to ensure sufficient material, resulting in four different biological repeats per plant line. The biological repeats were processed and analysed individually. The results are the average of these four biological repeats except for the GC–MS datasets where only three repeats were processed. All samples were analysed for statistical significance using Microsoft Excel 2016 and univariate statistical analyses were performed (ANOVA, with *p* = 0.05) under the guidance of the Centre for Statistical Consultation at Stellenbosch University (Prof. Martin Kidd) using Statistica 10 (StatSoft Southern Africa—Analytics, Sandton, South Africa). Py-GC–MS results were presented as variability plots after least significant difference (LSD) testing was performed. Multivariate analysis such as principal component analysis (PCA) was performed with the SIMCA 14 software package (Sartorium Stedim Biotech - Umetrics AB, Umea, Sweden).

## 3. Results

### 3.1. Lignin Compositional Analysis of Wild Type Versus Transgenic Tobacco Using Py-GC–MS

**Figure 1:**
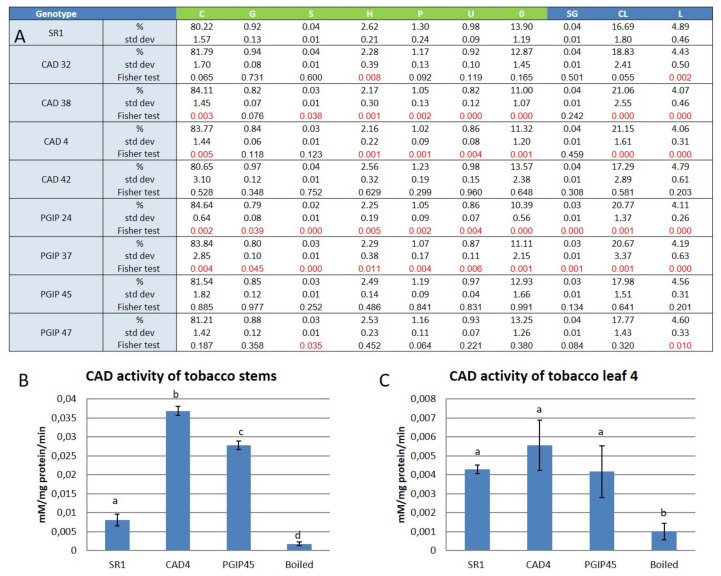
(**A**): Table of the lignin relative content and composition of the harvested tobacco leaves. Results are presented as the percentage of the total spectra recorded. Each percentage is the average of four biological repeats of four different leaves, showing standard deviation. A two-way ANOVA followed by a Fisher least significant difference (LSD) post-hoc test showed if the variations of the component analysed using Py-GC–MS between the different tobacco transgenic lines versus the wild type SR1 were significant with *p* ≤ 0.05. C: Carbohydrate related components; G: Guaiacyl; S: Syringyl; H: p-hydroxyphenol; P: generic phenolics; U: known spectra, unknown identification; O: unknown spectra; SG: Syringyl/Guaiacyl ratio; CL: Carbohydrate related components/Lignin ratio; L: total lignin. (**B**): CAD enzyme activity assay of tobacco stems performed over a 10 min period in triplicate and expressed as mM/mg protein/min. (**C**): CAD enzyme activity assay of tobacco leaf 4 performed over a 10 min period in triplicate and expressed as mM/mg protein/min. In B and C, data were obtained from three biological repeats and boiled samples are controls where samples were boiled to inactivate proteins prior to the test. A one-way ANOVA followed by a Tukey test were performed with *p* ≤ 0.05 to statistically separate the samples.

Pyrolysis gas chromatography mass spectrometry (Py-GC–MS), which allows thermogravimetric quantification, was used to determine the changes in the leaf lignin composition of transgenic tobacco plants compared to the wild type controls. Nine genotypes were tested: eight transgenic tobacco lines including four plants overexpressing the *NtCAD14* gene (these were named lines CAD4, CAD32, CAD38, and CAD42, according to the data presented in Mbewana [33] and four plants overexpressing the *VviPGIP1* gene (these were named lines PGIP24, PGIP37, PGIP45, and PGIP47; see Table 1). The *Nicotiana tabacum* cv Havana petit SR1 was used as the control line; and two sets of plants of SR1 were grown, one as controls for *NtCAD14* and one set of controls for *VviPGIP1* containing plants (see Table 1). In overview, the results from the Py-GC–MS analyses revealed that on average 80–85% of the spectra obtained from tobacco leaf AIR were made up of carbohydrates or carbohydrate related components (see Figure 1A). A further 10–14% of the spectra accounted for unknown compounds present in the AIR samples (Figure 1A). Only about 4% of the AIR recovered spectra was identified as known lignin components (Figure 1A). Little difference was observed for total lignin content and also in terms of the percentage of various lignin components identified between the different genotypes. In all the genotypes tested, p-hydroxyphenol was the most abundant monomer making up 2.1–2.6% of the total known lignin components, followed by generic phenolics (1–1.3%), guaiacyl (0.8–1%) and syringyl (0.02–0.04%). To ascertain if the enzyme activity for cinnamyl alcohol dehydrogenase (CAD) was equivalent between stems and leaves an experiment was conducted using representative samples, i.e., CAD4 for *NtCAD14* containing lines and PGIP45 for the *VviPGIP1* plants (see Figure 1B,C). Both transgenic lines displayed increased CAD activity in the stems (four to six times higher than SR1), whereas no such increased activity occurred in the leaves.

**Figure 2:**
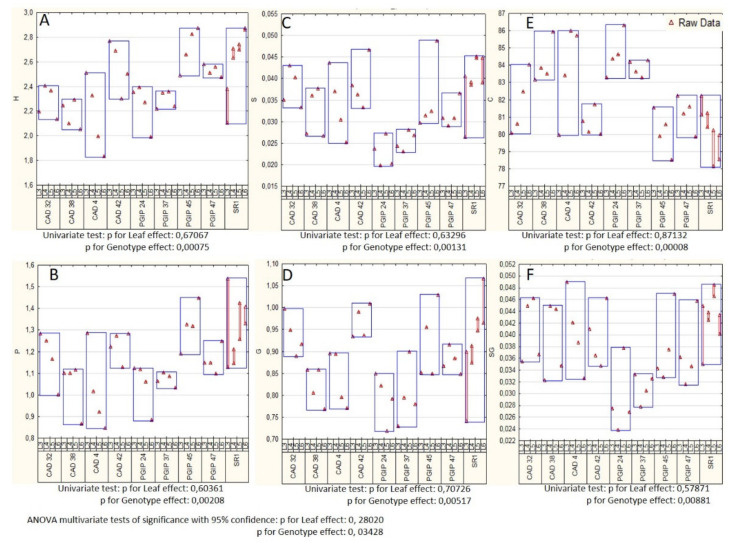
ANOVA box plots (multivariate tests of significance with 95% confidence intervals) of Py-GC–MS data for each analyte category SR1 vs transgenic plant lines. (**A**): p-hydroxyphenol (H); (**B**): generic phenolics (P); (**C**): syringyl (S); (**D**): guaiacyl (G); (**E**): carbohydrate related components; and (**F**): syringyl/guaiacyl ratio (SG). Data from SR1 constitute eight biological repeats whereas all other lines represent four biological repeats per transgenic plant.

A Fisher analysis was performed as well as ANOVA multivariate tests of significance were used to evaluate the differences observed between SR1 as control and all genotypes for all leaf positions (see Figure 1A and Figure 2; see also Figure S2 for Supplementary Materials). For example, the wild type SR1 and the transgenic lines CAD42 and PGIP45 showed no significant differences for any of the compounds assayed (i.e., p-hydroxyphenol, guaiacyl, syringyl, general phenolics, carbohydrate components and the syringyl to guaiacyl ratio (S/G ratio)). However, the PGIP24 and PGIP37 lines interestingly showed a reduction in the S/G ratio compared to the control plants. The other lines showed more variable responses with many being non-significant as compared to the control plants. However, lines PGIP24 and PGIP37 had significantly lower values for all the monolignols assayed compared to the wild type plants (Figure 2), whereas the CAD32 line had significantly less p-hydroxyphenol (Figure 2A) and PGIP47 had significantly less syringyl (Figure 2C) compared with SR1 plants. Considering previous research had focused on the arabinoxyloglucan component of *VviPGIP1* transgenic tobacco and since the AIR was 80% by weight primarily polysaccharide in nature it was logical to further investigate the polysaccharide–protein network of these transgenic plants in relation to their controls.

### 3.2. Monosaccharide Composition Analysis of Wild Type Versus Transgenic Tobacco Using GC–MS

**Figure 3:**
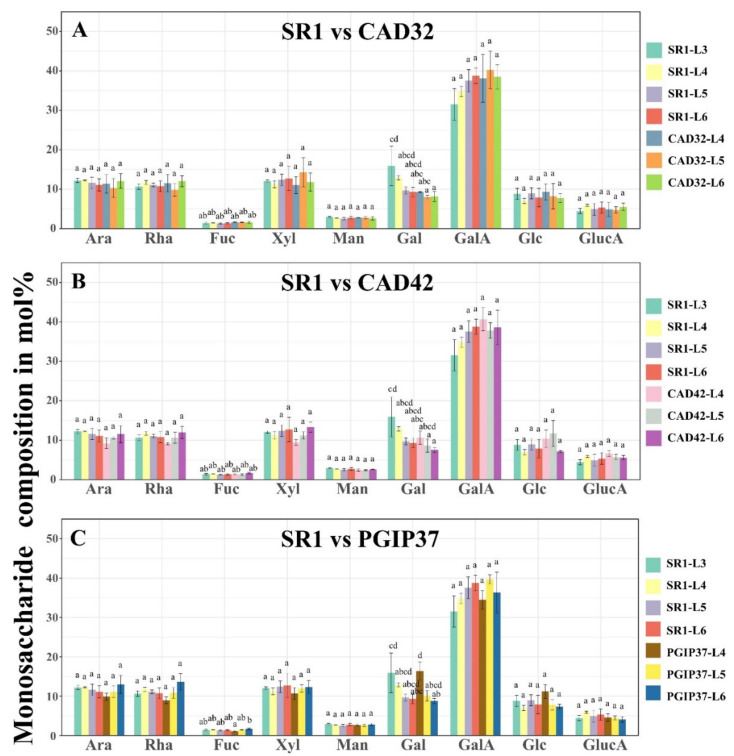

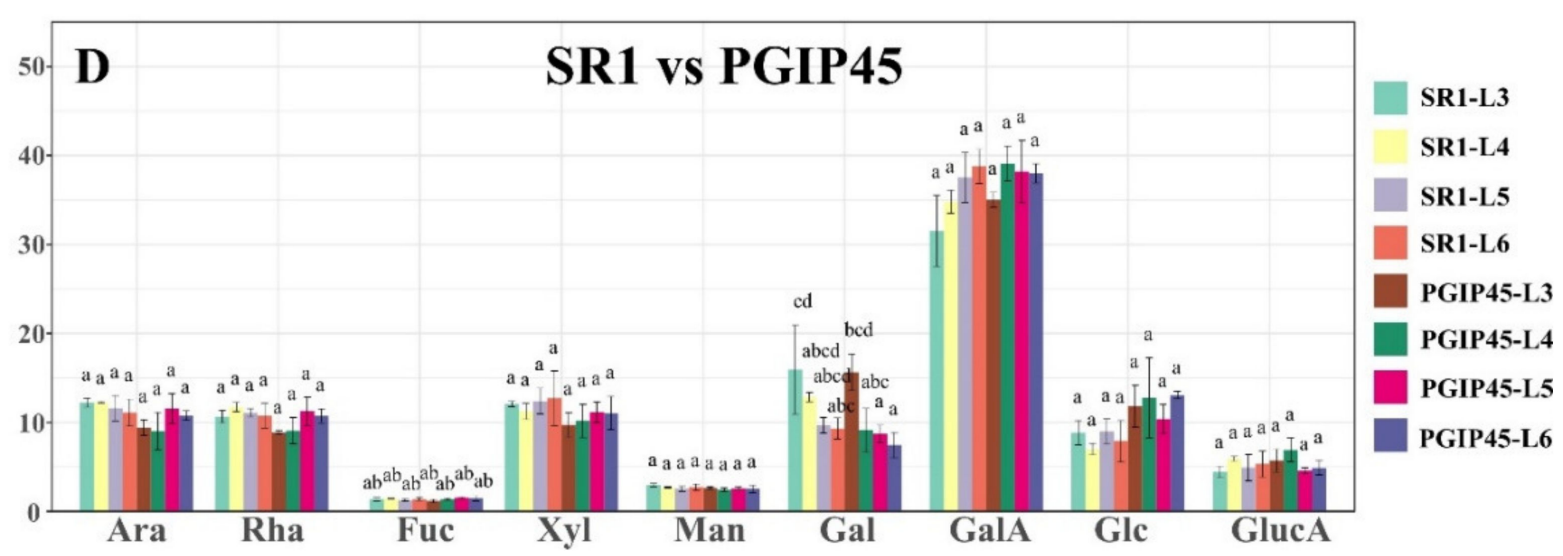
Monosaccharide composition of the cell wall, expressed in mol%, from the total alcohol insoluble residue (AIR) extraction of leaves from different tobacco lines. (**A**): Wild type SR1 versus CAD32. (**B**): SR1 versus CAD42. (**C**): SR1 versus PGIP37. (**D**): SR1 versus PGIP45. Ara: arabinose; Rha: rhamnose; Fuc: fucose; Xyl: xylose; Man: mannose; Gal: galactose; GalA: galacturonic acid; Glc: glucose; GlcA: glucuronic acid; L: leaf. The bars represent the average of three biological repeats. A two-way ANOVA followed by a Tukey’s test were performed to statistically separate samples with *p* ≤ 0.05.

Monosaccharide analysis of tobacco AIR yielded a general profile showing; ca. 10 mol% of arabinose (Ara), ca. 10 mol% of rhamnose (Rha), ca. 1–2 mol% of fucose (Fuc), ca. 10 mol% of xylose (Xyl), ca. 2.5 mol% of mannose (Man), ca. 8–15 mol% of galactose (Gal), ca. 32–40 mol% of galacturonic acid (GalA), ca. 7–10 mol% glucose (Glc), and ca. 5 mol% glucuronic acid (GlucA; see Figure 3). Only representative lines are shown; namely CAD32, CAD42, PGIP37, and PGIP45 compared to wild type SR1 tobacco; this pattern was repeated in all lines. For the wild type plants, a standard developmental pattern was observed from leaf position 3 (L3) to leaf position 6 (L6), which showed alterations in the levels of Gal and GalA (Figure 3). The Gal decreased in a staggered manner from ca. 15 mol% for L3 to ca. 9 mol% for L6 while the GalA levels increased from ca. 32 mol% for L3 to ca. 38 mol% for L6 (the trend was visible but not statistically significant). The values between the other monosaccharides were not significantly different. A similar developmental pattern for Gal was observed with the transgenic lines PGIP37 and PGIP45 where Gal levels dropped from ca. 15 mol% for L3 or L4 to ca. 8 mol% for L6. Overall no difference in the monosaccharide profiles was observed between transgenic (both CAD and PGIP lines) and wild type SR1 tobacco.

### 3.3. Comprehensive Microarray Polymer Profiling (CoMPP) of SR1 Versus CAD Transgenic Tobacco Lines

**Figure 4:**
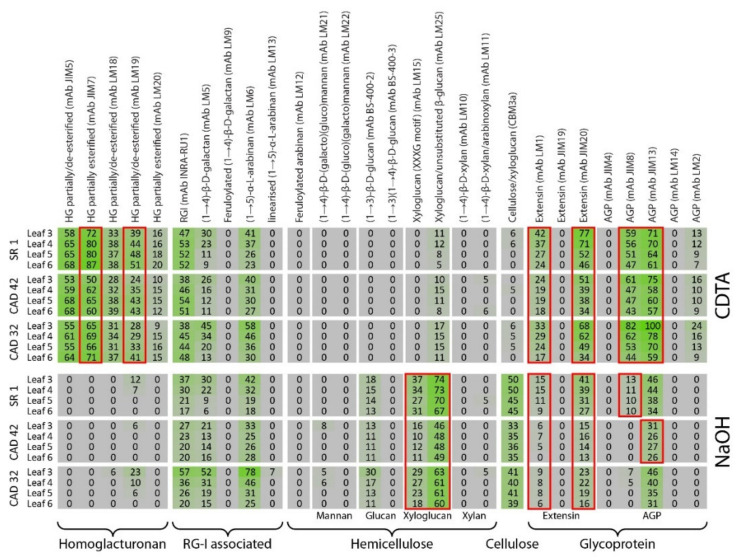
CoMPP (comprehensive microarray polymer profiling) of the wild type SR1, CAD42, and CAD32 tobacco leaf AIR. Heatmap showing cell wall polysaccharides and proteins relative abundance, which are from the average of four biological repeats.

In addition to total sugar composition analysis, an overview of the polysaccharides and glycoproteins present in the plant cell wall was obtained by performing a comprehensive microarray polymer profiling (CoMPP) analysis. A CoMPP analysis uses a range of monoclonal antibodies (mAbs) and carbohydrate binding modules (CBM) that recognise specific epitopes present on different cell wall polysaccharides. The CoMPP analysis of the CDTA extract from the CAD42 and CAD32 lines versus SR1 yielded a heatmap rich in pectins specifically in homogalacturonans (HGs) and rhamnogalacturonan I associated polymers (see Figure 4). Homogalacturonans (HG) are major pectin components. They are recognised by the monoclonal antibodies (mAbs) JIM5, JIM7, LM18, LM19, and LM20 (these bind depending on the degree and pattern of methylesterification of pectins). A leaf position (ontogenic or leaf age) pattern was observed with the wild type SR1 with an increase in signal for mAbs JIM5, JIM7, and LM19 from the youngest to the oldest leaf (Figure 4). The wild type SR1 had a stronger signal for mAbs JIM7 and LM19 than the CAD lines. Rhamnogalacturonan I (RG-I) is another major pectin polysaccharide; and the INRA-RU1 mAb binds to the RG1 backbone structure (alternating sequence of GalA and Rha monomers). Similarly to that found for HG epitopes, an increase in the INRA-RU1 signal, as a function of leaf maturation, was observed. The mAbs LM5 for galactans and mAbs LM6 for arabinans both showed a decreasing pattern of abundance and these are found as side chains of RG-1 polymers. In addition to polysaccharide binding mAbs, some probes were used that bound to cell wall glycoproteins. For the extensins, mAbs LM1, JIM19, and JIM20 were selected and for the arabinogalactan proteins (AGPs) mAbs JIM4, JIM8, JIM13, LM14, and LM2 were used. A similar decreasing signal as a function of the leaf position was notably found for mAb LM1, JIM20, JIM8, and JIM13 in all lines. However no (or very low) signals were found for mAbs JIM19, JIM4, LM14, and LM2 in the CDTA pectin fractions. It did seem however that important differences in younger leaves occurred with significantly more extensin signal (mAbs LM1 and JIM20) for the wild type compared with the CAD lines (i.e., CAD32 had higher signal than CAD42). For the AGPs it was the opposite with a much stronger signal for mAbs JIM8 and JIM13 in CAD32 than for CAD42 and SR1.

CoMPP analysis of the NaOH extract from the CAD42 and CAD32 lines versus SR1 yielded a heatmap rich in RG-1 polymers, hemicelluloses such as xyloglucan and cellulose; and glycoproteins (i.e., extensins and AGPs; see Figure 4). For the INRA-RU1 mAb a decreasing signal was observed as a function of leaf maturation. The mAb LM5 galactan and LM6 arabinan chains linked to the RG-I backbones showed a decreasing signal. The RG-1 and side chain signals were similar between wild type and transgenic lines, except for leaf position 3, which showed some variability in total signals. The other main polysaccharides are the hemicelluloses and cellulose-linked epitopes. In the tobacco leaf AIR, glucan (mAb BS-400-2) and xyloglucan (mAbs LM15, LM25) binding antibodies and CBM3a displayed a signal in the NaOH fraction. The BS-400-2 mAb recognises glucans but showed no significant change in SR1 versus CAD42, whereas a very slight decrease in signal was observed as a function of leaf maturation for CAD32. For the xyloglucans, there was a slight decrease in signal for the mAb LM15 that recognises the XXXG motif (LM15) while the mAb LM25 signal remained stable. The wild type had a stronger signal for the CBM3a, a xyloglucan-cellulose binding probe than the CAD lines, and its intensity remained constant between the different leaves. The wild type had stronger signals for xyloglucan probes than CAD32, which in turn had higher signal than CAD42. The mAbs LM12, LM21, LM22, and BS-400-3, which bind mannans and some glucan motifs did not show any (or a very low) signal. For the extensin probe JIM20 the signal was higher in the wild type than the transgenic. For the AGP probe mAb JIM13 the signal pattern was very similar between the wild type and the CAD lines. However the total signal concentration did vary being lower in the transgenics for both extensins (mAb JIM20) and AGPs (mAb JIM13).

**Figure 5:**
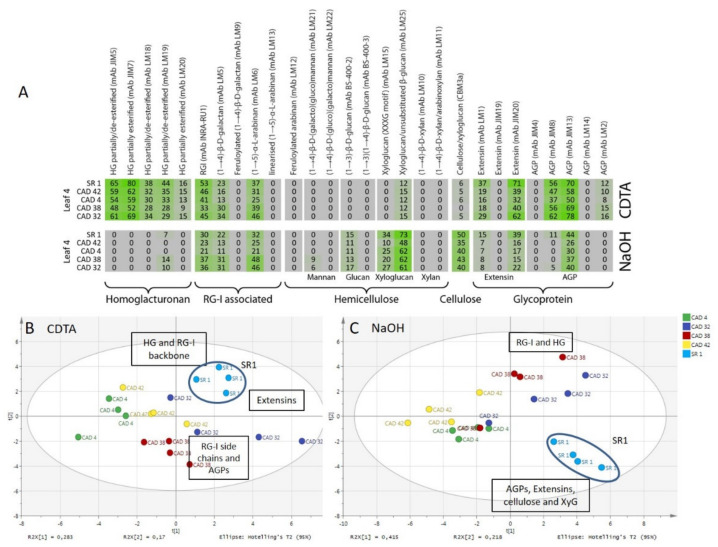
CoMPP of the leaf 4 AIR from SR1, CAD42, CAD4, CAD38, and CAD32 tobacco lines. (**A**): Heatmap showing cell wall polysaccharides and proteins relative abundance using antibodies signal intensity reads, presented results are the average of four biological repeats. (**B**): PCA of the pectin-rich fraction (CDTA) and (**C**): PCA of the hemicellulose-rich fraction (NaOH). On the PCA, samples are coloured according to their genotype. The wild type SR1 samples are highlighted and circled. Data obtained from four biological repeats.

Supporting datasets for CAD4 and CAD38 are provided (see Supplementary Materials Figures S3 and S4 with CAD4, CAD38, and SR1). It was decided, in order to simplify the interpretation, to focus on one leaf position only (see Figure 5). L4 or leaf position 4 was chosen as an intermediate developmental stage; whereas L3 leaf position 3 showed quite a lot of sample variation across batches. A simplified heatmap was constructed for L4 from datasets for CDTA and NaOH (see Figure 5), effectively removing the ontogenic patterning. Compared to the CAD4, CAD42, CAD32, and CAD38 lines, the wild type had a stronger signal for homogalacturonan binding mAbs JIM5 and JIM7, which target HGs displaying both low and high degrees of methylesterification. No clear patterns between wild type and transgenic was found for the RG-1 associated epitopes (e.g., mAbs INRA-RU1, LM5, and LM6), although CAD38 and CAD32 seemed to display higher signals for mAbs LM5 and LM6 compared to the other transgenics and the wild type. The probes LM25 and CBM3a that recognise xyloglucan epitopes both had higher signal intensity in the NaOH extracts of the wild type compared to the transgenics. The most prominent differences between wild type and transgenic lines were for the extensin and some of the AGP epitopes (i.e., cell wall proteins). The data indicated that for the extensin probes mAb JIM20 and LM1, higher signal values were found for the wild type plants. Similarly for mAb JIM13 (an AGP probe) higher values were found for the wild type plants in the NaOH fraction. This is confirmed in the PCA plots for CDTA (Figure 5B) and NaOH (Figure 5C) extracts where SR1 separated from the transgenic populations due to higher signal values for cell wall proteins.

### 3.4. Comprehensive Microarray Polymer Profiling (CoMPP) of SR1 Versus Grapevine PGIP1 Transgenic Tobacco Lines

**Figure 6:**
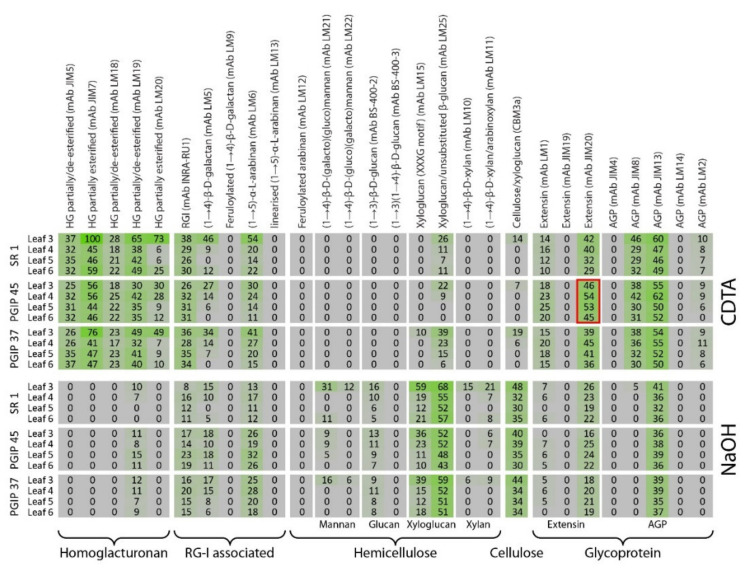
CoMPP of the wild type SR1, PGIP37, and PGIP45 tobacco leaf AIR. Heatmap showing cell wall polysaccharides and proteins relative abundance using antibodies signal intensity reads, presented results are from the average of four biological repeats.

CoMPP analysis of the SR1 control versus the four transgenic lines PGIP24, PGIP37, PGIP45, and PGIP47 yield a profile very similar to the preceding analysis on CAD transgenics although the distinction between wild type and transgenic is more subtle for the PGIP lines (see Figure 6; Figure S5). Similarly to the procedure followed for the CAD lines, PGIP24 and PGIP47 were excluded from this analysis as being very similar to PGIP37 and PGIP45 but the figures are available as supplementary data (Figures S5 and S6). The most conspicuous difference on first inspection was the significant difference between leaf 3 and the other leaf positions (Figure 6). Much higher signal intensities were found in the CDTA fraction for leaf 3 for mAbs JIM7, LM19, LM20, LM5, LM6, and LM25 compared to the other leaf positions. These probes bound to partially and highly methylated homogalacturonan, galactan, arabinan, and xyloglucan respectively. For leaf positions 4–6, signal variations were not observed except for the xyloglucan binding mAb LM25, which showed a decreasing signal with leaf maturation. In general, the mAbs showed similar signal intensities between the three lines except for mAb JIM20 (the extensin probe), which yielded a slightly stronger signal for the PGIP45 line compared to the wild type (Figure 6). The pattern was repeated for the NaOH extract where leaf position 3 separated markedly from the leaf 4–6 samples (Figure 6). This appeared to be due to stronger signals for the mAbs LM21, LM15, LM25, and CBM3a, which bind to mannan, xyloglucan, and cellulose epitopes (Figure 6).

**Figure 7:**
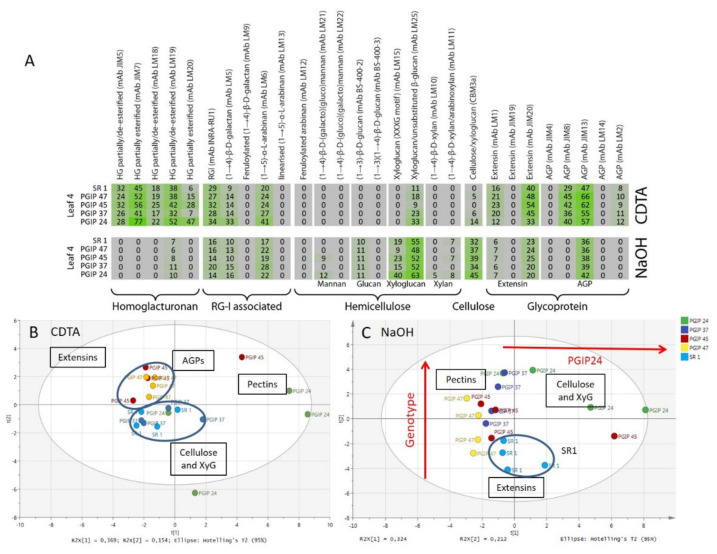
CoMPP of the leaf 4 AIR from SR1, PGIP24, PGIP37, PGIP45, and PGIP47 tobacco lines. (**A**): Heatmap showing cell wall polysaccharides and proteins relative abundance using antibodies signal intensity reads from the average of four biological repeats. (**B**): PCA of the pectin-rich fraction (CDTA) and (**C**): PCA of the hemicellulose-rich fraction (NaOH). On the PCA, samples are coloured according to their genotype. The wild type SR1 samples are highlighted and circled.

As performed for the analysis of the CAD lines, heatmaps were constructed using only the CDTA and NaOH datasets of the leaf 4 samples from the SR1 and PGIP lines (see Figure 7). In the CDTA extracts signals for the common pectin probes mAbs JIM5, JIM7, LM18, and LM19 were found and were relatively invariant between wild type and the PGIP lines. Similarly the INRA-RU1 and LM6 probes did not show much change in the CDTA fractions. Interestingly, especially for the lines PGIP47 and PGIP45, the signal intensities for mAb LM1, JIM20, JIM8, and JIM13 (extensins and AGPs) were found to be markedly higher in the pectin CDTA fraction compared to the wild type plants. In contrast for PGIP lines 24, the pectin signals (mAbs LM19, INRA RU1, LM5, and LM6) and LM6 for PGIP line 37 were found to be higher in the transgenics for the NaOH fraction.

## 4. Discussion

### 4.1. Lignin Composition was Mostly Unaltered in Leaves of Plants Overexpressing Either CAD or the Grapevine PGIP1 Gene

The lignin analysis performed showed no significant difference in the total lignin concentration in the leaf as a function of leaf age, although ANOVA showed that significant differences did occur at the genotype level. This correlates with the results from Alexandersson et al. [18] that showed that *Vvipgip1* expression induced upregulation of CAD but that differences in the total lignin content of the leaf was not significant. Similarly, Mbewana [33] showed that tobacco plants overexpressing *CAD* had higher CAD activity in stems, but no significant differences in CAD activity were found in leaves when compared to the wild type. This indicated that overexpression of *CAD* was localised to the stem where lignin accumulation occurred with little to no effects at the leaf level. Overall, CAD activity was very low in leaves compared to the stems and this makes sense in the context of leaf function as a rapidly growing organ maximising surface area for photosynthesis. Increased levels of leaf lignification would probably be detrimental as it would impact leaf growth-expansion and therefore *CAD* expression is probably highly regulated in leaves. This argument is supported by observations of the CAD4 line, which had the highest lignin levels of all the tobacco overexpressing *CAD* lines generated, which shows a delayed and slow growing phenotype compared with other plants. This means that lignification must be tightly controlled for the plant to achieve normal growth and development [66]. The PGIP tobacco lines, similarly to the CAD lines, had an elevated CAD activity in stems but not in the leaves compared to SR1. Those results tend to confirm the model proposed by Nguema-Ona et al. [34] with spatial separation of lignification in PGIP-induced *CAD* expressing plants. CAD is a key enzyme in conversion of cinnamyl aldehydes to alcohols, which is the final step in monolignol biosynthesis before polymerisation [67]. Thus, *Vvipgip1* and *CAD* upregulation may not necessarily affect lignin quantity, but lignin composition might well be altered. It is interesting to note that PGIP24 and PGIP37, which have the highest increase in resistance to *Botrytis cinerea* compared to the wild type [24], had significant differences in all the monolignols analysed while PGIP45 and PGIP47, which displayed lower resistance to the necrotrophic fungus, had a lignin composition much closer to SR1. The CAD4 line, which had the lowest acquired resistance to *B. cinerea* among the CAD lines tested [33], was also the one that differed the most from SR1. Since the concentration of lignin is not the determining factor, we investigated if the ratio between different monolignols could be responsible for observed resistance phenotypes. Indeed higher levels of syringyl units have been linked to higher resistance to pathogen attacks [68,69,70]. However, none of the lines tested had more syringyl than the wild type and the ratio of syringyl with the other monolignols (i.e., the S/G ratio) have not in all cases been correlated with an increased resistance to biotic stress [68]. This is in line with our results where PGIP24 and PGIP37, the less susceptible lines to *B. cinerea*, had the lowest S/G ratio. These data alone were insufficient to support the hypothesis that lignin changes prior to the infection are responsible for the increased resistance phenotype to *B. cinerea* infection. It is necessary to also look at the polysaccharide and protein components of the leaf cell walls to search for cell wall architectural changes linked to resistance priming against pathogenic fungi.

### 4.2. Pectin Organisation Varied between the SR1 and the Transgenic CAD and PGIP Lines

The CoMPP analysis performed on both CAD and *Vvi*PGIP1 transgenic plants showed differences in polysaccharide availability due to the leaf maturation stage and age. Large variations within the leaf 3 sample repeats were found as compared to other the leaf positions 4–6. Leaf 3 leaves are still growing and have not fully matured. Size variations, due to non-perfectly synchronised plants, might have brought about these observable differences. To assess this potential problem, we analysed leaves separately and characterised the ontogenic effect. The ontogenic effects linked to the leaf maturation process include a reduction in the galactan and arabinan side chains associated with RG-I as the leaf matures. Such changes associated with RG-1 side chains have been well described in the literature [71,72] and involve cell elongation and cell wall tightening events. These pectin side chains have been shown to be linked to cellulose microfibrils [73,74] and support the xyloglucan/cellulose network [75]. The decreasing xyloglucan probe signals found for many of the leaf CDTA fractions, which is probably linked to the arabinan and galactan side chains helping form pectin-xyloglucan linkages, supports this. In addition to its role in the lignin biosynthesis pathway, *CAD* seems to be directly and/or indirectly involved in cell wall polysaccharide reorganisation. In datasets from the CAD lines it seemed that xyloglucan and cellulose had become less accessible as compared to the SR1 lines suggesting biosynthetic alterations and/or architectural reorganisation. A role for xyloglucan changes has already been well established in PGIP lines. The downregulation of *XTH/XET* gene expression in *Vvi*PGIP1 plants [18] was further confirmed by Nguema-Ona et al. [34] who proposed a model where the cellulose/xyloglucan network was strengthened or ‘primed’ against pathogen attack in PGIP lines. A role for xyloglucan in functioning as the damage associated molecular pattern (DAMP) in plant–pathogen interactions supports this [35]. How *CAD* is able to directly influence *XTH/XET* gene expression remains unclear. Therefore, by lowering accessibility to the xyloglucan and cellulose network [2,76,77,78], the accessibility of the matrix to cellulases and other CWDEs would be substantially diminished.

A range of homogalacturonan binding mAbs were used, which displayed specificity to a variety methylesterified states or motifs. SR1 appeared to have the highest level of highly methylesterified HG. Although differences between SR1, CAD, and PGIP were slight and subtle; it would seem that PGIP lines seemed to maintain a stable degree of methylesterification of their leaf pectins. Pectin methylesterification protects pectin against the action of CWDEs and pectinases such as ePGs more specifically. The action of ePGs is essential for necrotrophic fungi such as *Botrytis* spp. to breach the cell wall barrier and penetrate plant tissues [10,11]. Methylesterification of the galacturonic acid residues from homogalacturonan can protect the plant against fungal pathogen as shown by Volpi et al. [79] in wheat by the overexpression of a pectin-methyl-esterase inhibitor (PMEI) from kiwifruit (see also the review from Lionetti et al. [80]). Homogalacturonan is synthesised in the methylesterified state and only demethylesterified in the cell wall by pectin-methyl-esterases (PMEs) [81,82]. During de-esterification they form homogalacturonan Ca^2+^ ion egg-box structures [83,84]. De-esterification of the transgenic lines HG polymers could facilitate the easier release of oligogalacturonides (OGs) into the apoplast during infection. Oligogalacturonides have been studied for their role as defence elicitors or DAMPs [29]. OG DAMPs would trigger other plant defence mechanisms, which could act as defence primers [26,27,28,30,31]. It is tempting to speculate that PGIP expression can modulate pectin esterification levels through signalling pathways so as to ‘prime’ the wall to be able to release OG DAMPs upon infection by a fungal pathogen allowing the plant to respond in a faster and stronger manner.

### 4.3. Extensin and AGP Epitope Distribution Varies between Wild Type and Transgenic Lines Suggesting a Role in Cell Wall Resistance

*Vvipgip1* overexpression seemed to elevate the level of extensins and AGPs in some of the lines. Extensins as proteins from the HRGP family have been more extensively studied in defence [85] and their role as cell wall strengthening agents is well documented [86]. Interestingly, Boudart et al. [87] showed that ePG action was able to induce extensin accumulation via a mechanism of OG elicitation. That process could also be involved with *Vvipgip*1 overexpression datasets presented. This could suggest that one of the priming defence mechanisms associated with *Vvipgip*1 overexpression is an elevated level of extensins. Cell wall proteins have been linked with a number of biotic (pathogen linked) or abiotic stress studies in plants [85,88,89]. AGPs have known roles in plant growth and development [89,90]. Mareri et al. [91] recently reviewed a role for AGPs in plant defence against biotic and abiotic stress. The authors reported AGP accumulation and up/downregulation of AGP encoding genes in plants have been observed during a range of abiotic stresses such as temperature stress, drought, flooding, hypoxia, salinity stress, mineral deficiency, and mineral toxicity. Additional findings have shown an accumulation of AGPs in root border cells have suggested they play an important role in recognition within the rhizosphere microbiome [92]. A more direct argument for an AGP role against biotic stress was found in pea roots where it was shown that in vitro, AGPs attract zoospores, inhibit cyst germination, and further mycelium development of *Aphanomyces euteiches* [93].

## 5. Conclusions

The overexpression of CAD and PGIP1 tobacco plants led to modifications of their cell wall structure in uninfected plants. Previous research has confirmed a role for arabinoxyloglucan modification of transgenic tobacco leaves, which could lead to a tightened cell wall matrix and the release of xyloglucan oligomers during infection, which could in turn act as DAMPs. The lignin composition in the PGIP lines (the S/G ratio specifically) did seem to correlate with resistance susceptibility however direct causality is lacking. At the level of pectin methylesterification the differences between transgenic lines and wild type was slight. Clearly, a more detailed picture of pectin methylesterification levels and patterning, by performing a more advanced analysis of pectins, is needed and could provide valuable new information on how the accessibility of HGs to ePGs in these transgenic lines is altered. Finally, cell wall proteins such as extensins and AGPs were modified by expression of both *NtCAD14* and *VviPGIP1* in tobacco plants. Further research should be focused on investigating what are the changes at the cell wall level in PGIP transgenic lines during an actual infection process with a necrotrophic pathogen such as *Botrytis cinerea*. Understanding the *in planta* functions of PGIP is a high priority area and this study has shed some valuable light in this respect; as well as in the area of ‘priming’ the plant cell wall for pathogen defence.

## Figures and Tables

**Table 1:** Table summarizing known information on the transgenic lines used in this study.

Plant Line	Promotor	Percentage of Successful Infections per Plant Line	Average Lesion Diameter (mm) *	Percentage Decrease in Disease Susceptibility (Compared to SR1) *^,^ **	Experiments Conducted on Those Lines	Reference
SR1	-	97%	24.0 ± 7.3 (11 dpi)	0		
CAD 4	CaMV 35Sp	93%	17.7 ± 5.2	27%	CAD enzyme activity assay	
CAD 32		93%	14.5 ± 3.4	40%	Whole plant infection assay	[33]
CAD 38		-	-	-		
CAD 42		97%	16.4 ± 3.6	32%		
SR1	-	75%	40.94 ± 3.5 (15 dpi)	0	Detached leaves and whole plant infection assay	[24]
					Gene expression analysis on infected and uninfected leaf tissue On uninfected leaves:	[18]
					XTH activity	
					Phytohormones analysis	
					Lignin staining	[36]
PGIP 24	CaMV 35Sp	83%	15.43 ± 0.9	62%	Monosaccharide composition	
PGIP 37		83%	12.60 ± 1.1	69%	Volatile organic compound analysis	
PGIP 45		92%	21.84 ± 3.1	47%	CoMPP	[34]
PGIP 47		92%	36.77 ± 3.2	10%	Arabinoxyloglucan analysis	

* Calculated 11 dpi for the CAD lines and 15 dpi for the VviPGIP1 lines. ** The decrease in disease susceptibility was calculated by comparing the average lesion diameter at 11 dpi for the CAD lines and 15 dpi for the VviPGIP1 lines to that of the untransformed control (SR1).

**Table 2:** List of monoclonal antibodies (mAbs) and CBMs used for the comprehensive microarray polymer profiling (CoMPP) analysis.

Homogalacturonan		low DE	JIM5	[43] [44]
		high DE	JIM7	
		Partially ME	LM18	[45]
		Partially ME	LM19	
		Partially ME	LM20	
RG-I associated		Backbone of RG-I	INRA-RU1	[46]
		D-galactan	LM5	[47]
		Feruloylated galactan	LM9	[48]
		L-arabinan	LM6	[49]
		Linearised L-arabinan	LM13	[50]
Hemicellulose		Feruloylate polymer	LM12	[51]
	Mannan	(galacto)(gluco)mannan	LM21	[52]
		D-(gluco)mannan	LM22	
	Glucan	β-D-glucan	BS-400-2	[53]
		Mixed link β-D-glucan	BS-400-3	
	Xyloglucan	Xyloglucan (XXXG motif)	LM15	[54]
		Xyloglucan	LM25	[51]
	Xylan	β-D-xylan	LM10	[55]
		β-D-xylan/arabinoxylan	LM11	
Cellulose		Cellulose (crystalline)	CBM3a	[56]
Proteins	Extensins		LM1	[57]
			JIM19	[58] [59]
			JIM20	
	AGP		JIM4	[60] [61]
			JIM8	[62]
			JIM13	[63] [61]
			LM14	[64]
		β-linked GlcA	LM2	[65]

## Supplementary Materials

The following are available online at https://www.mdpi.com/2076-393X/8/3/388/s1. Figure S1: A photograph of an SR1 tobacco plant used in this study in order to indicate leaf positions harvested (indicated by the numbered boxes). Out of the eight leaves present on the plant at the time of the experiment, leaf 2 was the youngest of those harvested, closest to the apical meristem and leaves 4-6 are fully expanded leaves. Figure S2: ANOVA box plots (multivariate tests of significance with 95% confidence intervals) of Py-GC-MS data for each analyte category SR1 vs transgenic plant lines. A: known spectra, unknown identification (U); B: unknown spectra (O); C: total lignin (L); D: Carbohydrate related components/Lignin ratio (CL). Each percentage is the average of four biological repeats of four different leaves, showing standard deviation. Figure S3: CoMPP of the CDTA fraction of the wild type SR1, CAD4 and CAD38 tobacco leaf AIR. Heatmap showing cell wall polysaccharides and proteins relative abundance using antibodies signal intensity reads which are the average of four biological repeats. Figure S4: CoMPP of the NaOH fraction of the wild type SR1, CAD4 and CAD38 tobacco leaf AIR. Heatmap showing cell wall polysaccharides and proteins relative abundance using antibodies signal intensity reads which are the average of four biological repeats. Figure S5: CoMPP of the CDTA fraction of the wild type SR1, PGIP24 and PGIP47 tobacco leaf AIR. A: Heatmap showing cell wall polysaccharides and proteins relative abundance using antibodies signal intensity reads which are the average of four biological repeats. B: PCA of the pectin-rich fraction (CDTA) C: Loading plot showing the separation by variables. On the PCA, samples are coloured according to their leaf position, each genotype is represented with a different symbol, and SR1 is circled. Figure S6: CoMPP of the NaOH fraction of the wild type SR1, PGIP24 and PGIP47 tobacco leaf AIR. A: Heatmap showing cell wall polysaccharides and proteins relative abundance using antibodies signal intensity reads which are the average of four biological repeats. B: PCA of the pectin-rich fraction (NaOH) C: Loading plot of the previous PCA showing the separation of the variable. On the PCA, samples are coloured according to their leaf position, each genotype is represented with a different symbol, and SR1 circled.
