# Supplementary figures and images for: Overexpression of VviPGIP1 and NtCAD14 in Tobacco Screened Using Glycan Microarrays Reveals Cell Wall Reorganisation in the Absence of Fungal Infection
Source: Vaccines (Basel). 2020 Jul 15;8(3):388. doi: 10.3390/vaccines8030388 (PMC7565493; doi:10.3390/vaccines8030388)

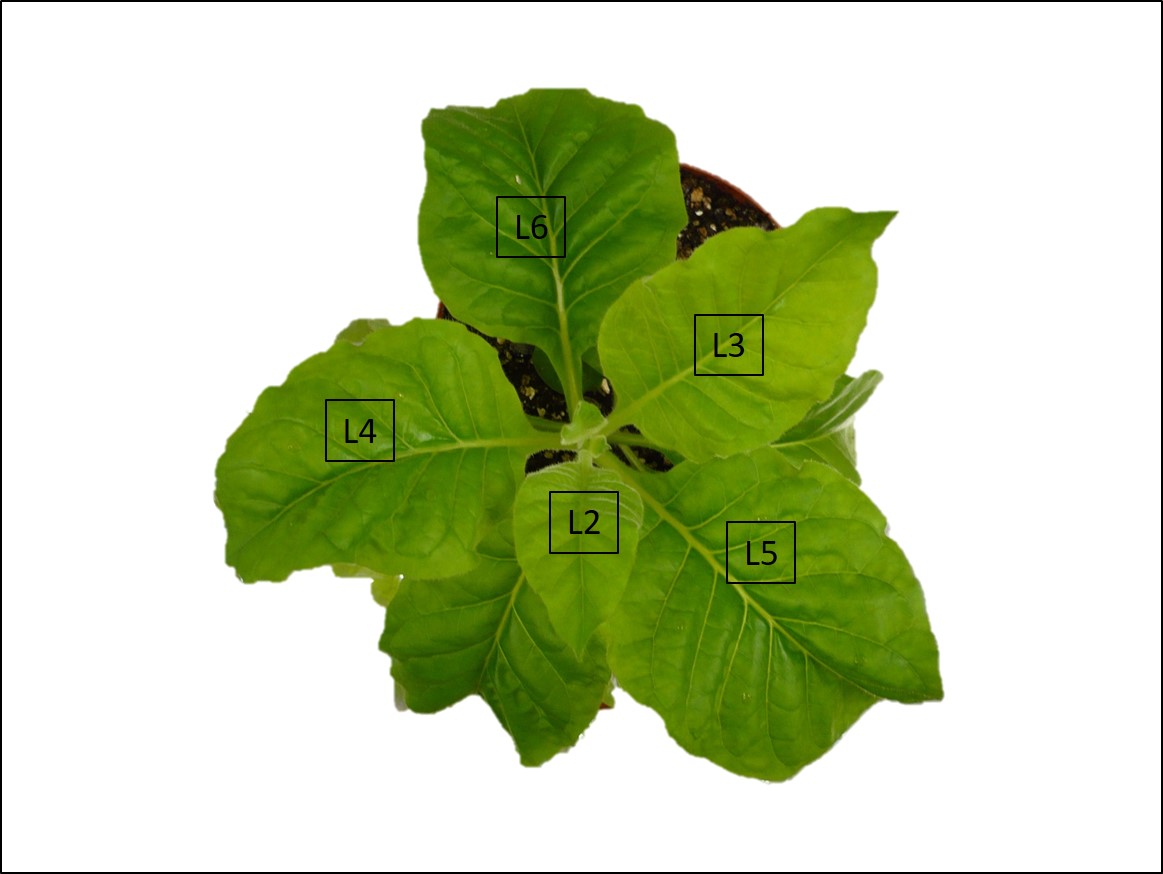

Supplement: Supplementary file 1 [file vaccines-08-00388-s001.zip › Figure S1.jpg]

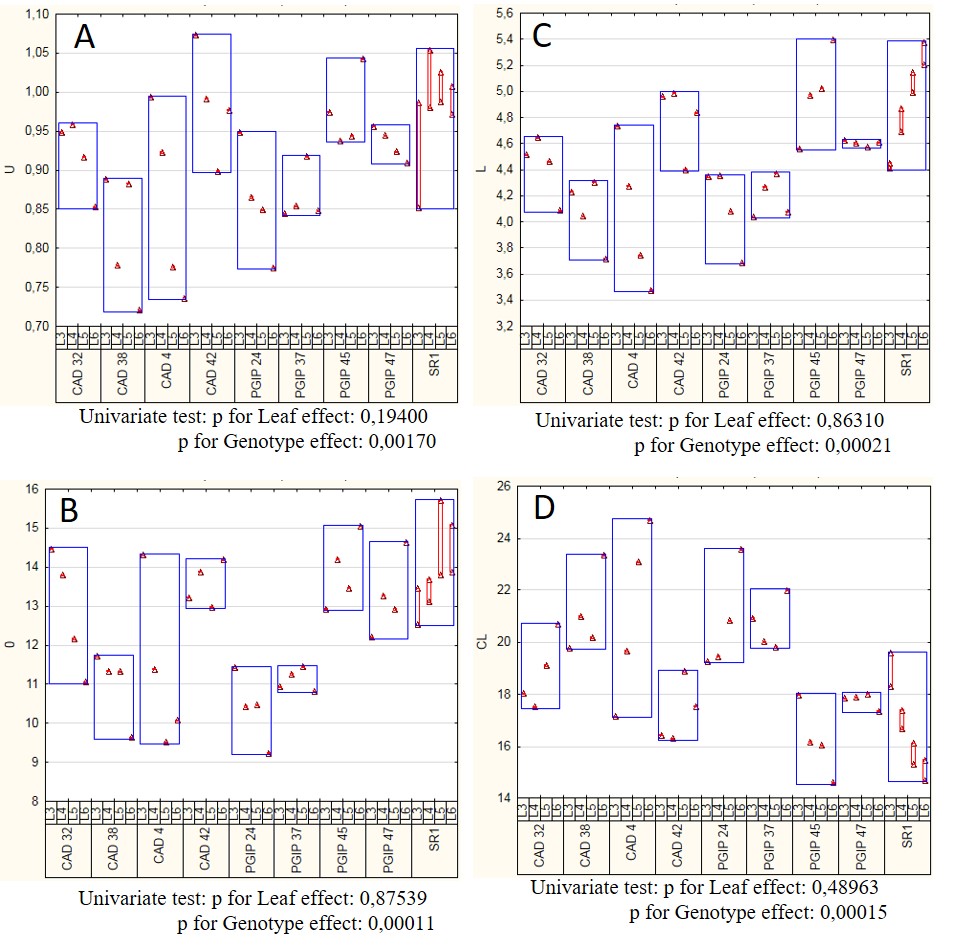

Supplement: Supplementary file 1 [file vaccines-08-00388-s001.zip › Figure S2.jpg]

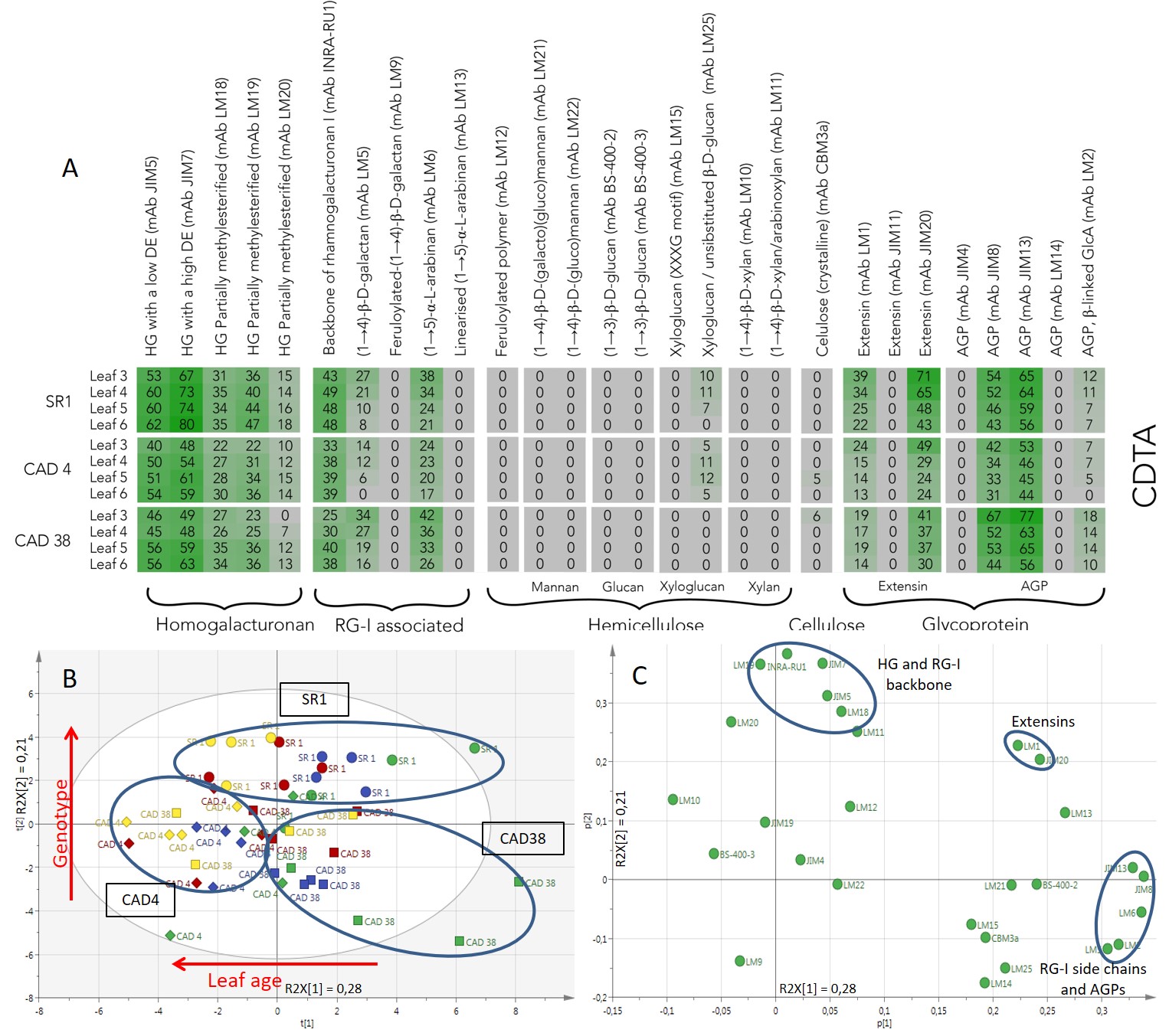

Supplement: Supplementary file 1 [file vaccines-08-00388-s001.zip › Figure S3.jpg]

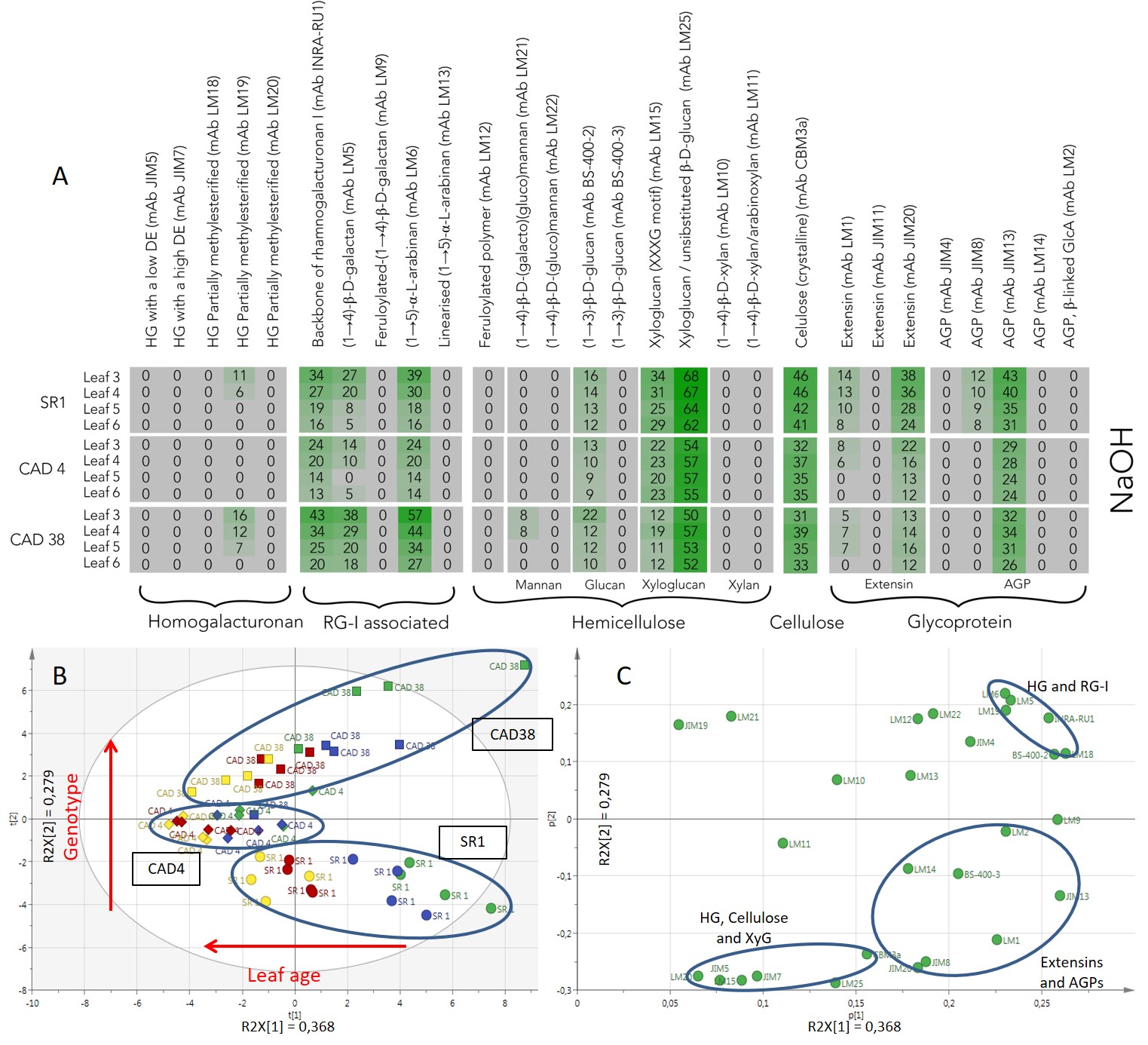

Supplement: Supplementary file 1 [file vaccines-08-00388-s001.zip › Figure S4.jpg]

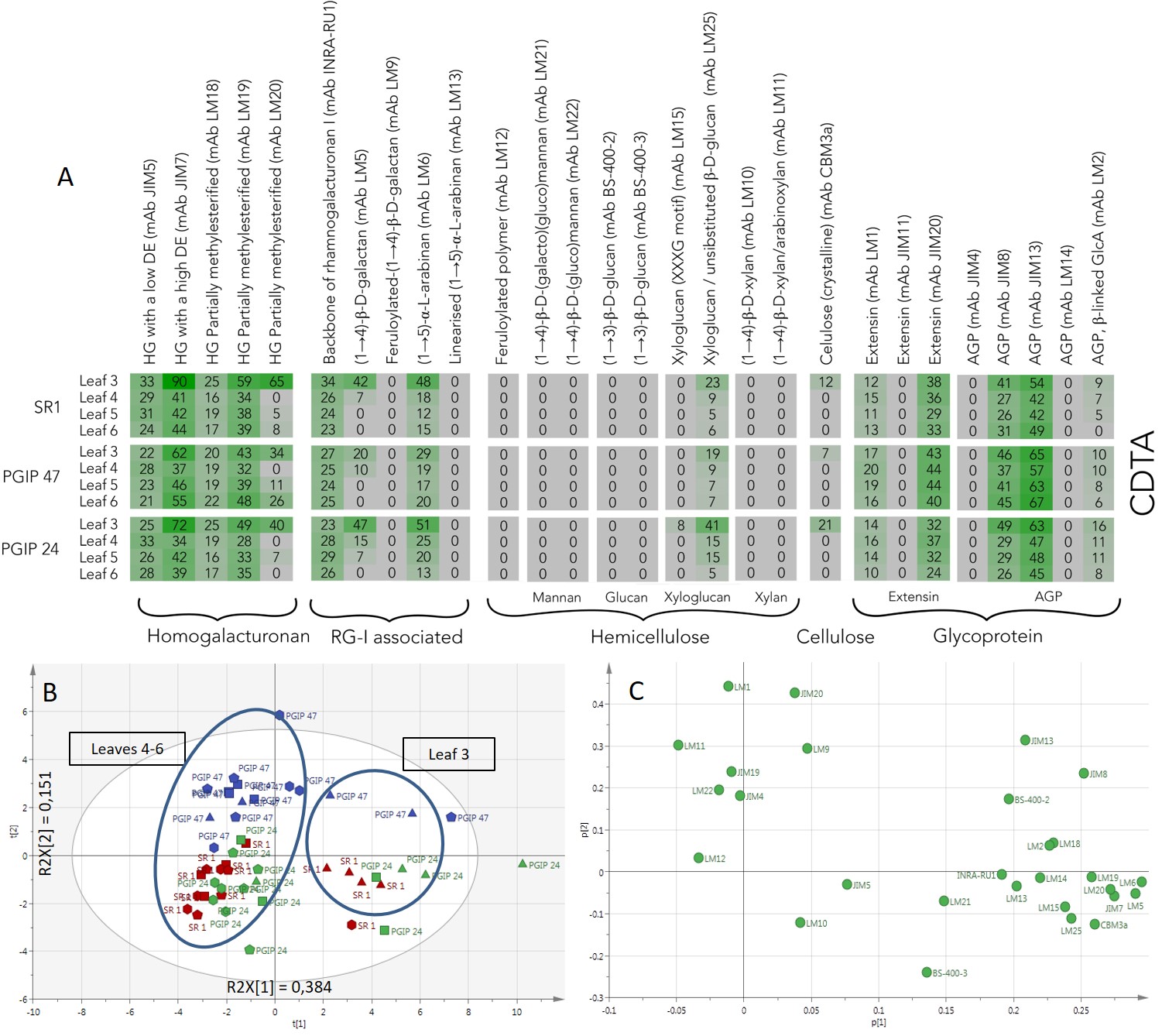

Supplement: Supplementary file 1 [file vaccines-08-00388-s001.zip › Figure S5.jpg]

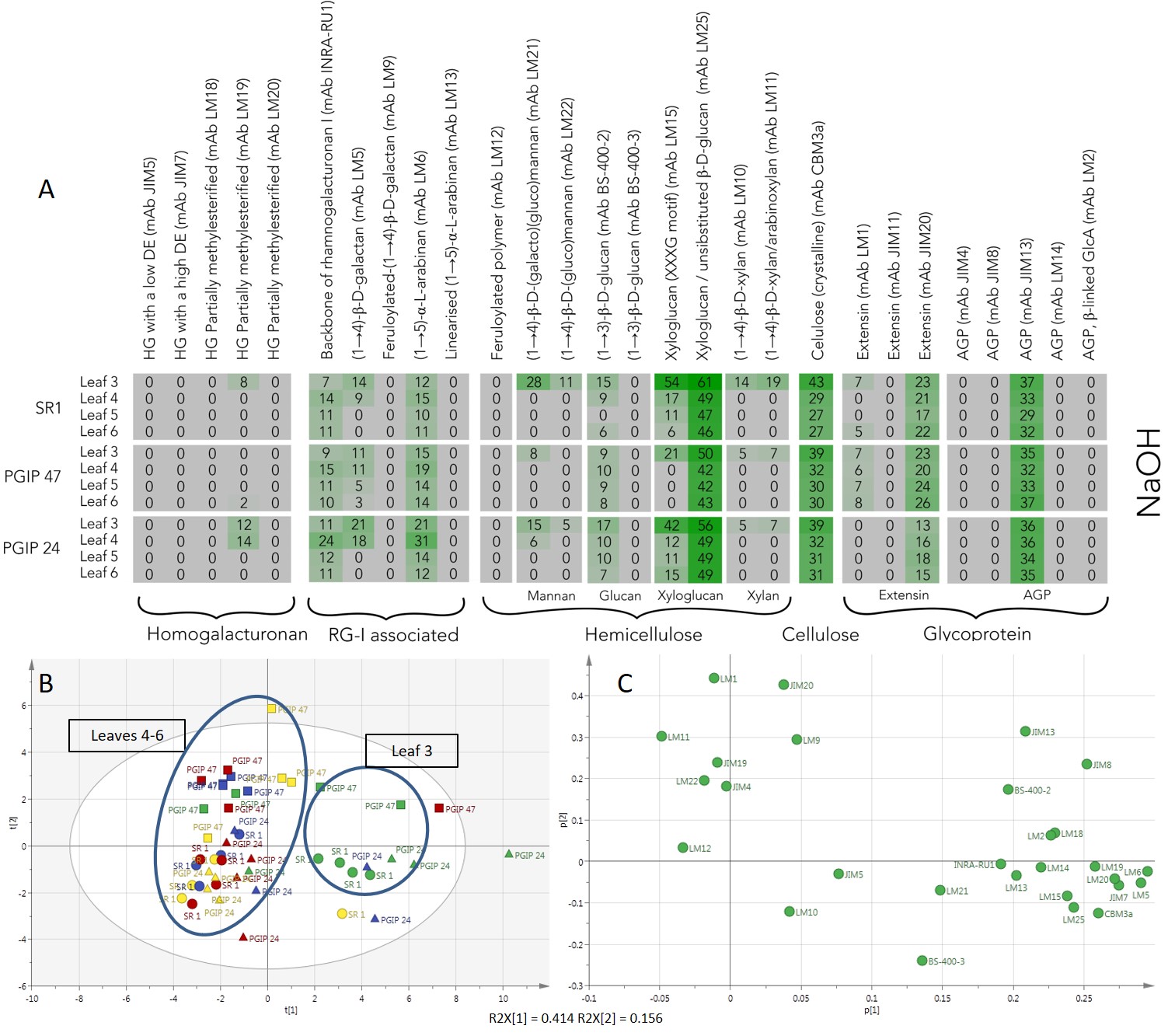

Supplement: Supplementary file 1 [file vaccines-08-00388-s001.zip › Figure S6.jpg]
